# Maraba virus-vectored cancer vaccines represent a safe and novel therapeutic option for cats

**DOI:** 10.1038/s41598-017-15992-4

**Published:** 2017-11-16

**Authors:** Jeff Hummel, Dorothee Bienzle, Annette Morrison, Michelle Cieplak, Kyle Stephenson, Josepha DeLay, J. Paul Woods, Brian D. Lichty, Byram W. Bridle

**Affiliations:** 1 McMaster Immunology Research Centre, McMaster University, Hamilton, Ontario, L8S 4L8 Canada; 2 CANSWERS, Clinical Trial Division, Georgetown, Ontario, L7G 5L8 Canada; 3 Department of Pathobiology, Ontario Veterinary College, University of Guelph, Guelph, Ontario, N1G 2W1 Canada; 4 Central Animal Facility, University of Guelph, Guelph, Ontario, N1G 2W1 Canada; 5 Animal Health Laboratory, University of Guelph, Guelph, Ontario, N1G 2W1 Canada; 6 Department of Clinical Studies, University of Guelph, Guelph, Ontario, N1G 2W1 Canada

## Abstract

Direct killing of malignant cells combined with induction of tumour-specific immune responses makes oncolytic vaccines attractive for cancer therapy. We previously developed a heterologous cancer immunization strategy that utilized a replication-defective adenovirus-vectored primary vaccine encoding a tumour antigen followed by boosting with a replication-competent Maraba virus expressing the same antigen. To assess the safety of oncolytic Maraba virus-based booster vaccines and inform the design of clinical trials, we conducted translational studies in cats, which have immune systems that are similar to people and spontaneously develop cancers of comparable types and etiologies. A dose of Maraba virus up to 2.5 × 10^11^ pfu per cat was well-tolerated, with adverse effects limited to mild, transient pyrexia, weight loss, neutropenia, lymphopenia and thrombocytopenia. Maraba viral genomes were present in some urine, stool and most plasma samples up to one week post-infection, but no infectious viruses were recovered. Post-mortem analysis showed one heart, one lung and all spleen samples contained Maraba virus genomes. No replication-competent viruses were recovered from any tissues. Post-mortem histopathological analyses revealed hyperplasia of lymphoid tissues, but no abnormal lesions were attributed to vaccination. This study demonstrated that Maraba virus-vectored cancer vaccines were well-tolerated and supports their use in treating cats.

## Introduction

To meet the need for more efficacious and targeted treatments for cancers, a growing number of oncolytic viruses (OVs) are being developed as vaccines to induce tumour-specific immune responses^1^. Oncolytic viruses preferentially replicate in cancer cells and effectively kill them either through lysis or induction of apoptosis. Although most traditional vaccines consist of non-replicating viruses, the use of replicating OVs as vaccine vectors has enhanced therapeutic benefits in animal models. Conceptually, OVs combine the benefits of direct killing of tumour cells with induction of tumour-specific immune responses and reversal of local immunosuppression, particularly when heterologous prime-boost vaccine strategies are applied^2–4^. Historically, prime-boost vaccinations were homologous in nature and involved re-administration of the same vaccine vector to enhance pathogen-specific responses. More recently, cancer vaccine studies have revealed that prime-boost vaccinations can be given using the same transgene (*e*.*g*. a tumour antigen) delivered by different vaccine vectors in a heterologous fashion. This is essential when developing oncolytic vaccines because homologous vaccination leads to preferential boosting of immune responses against highly immunogenic proteins derived from the OV backbone, thereby compromising secondary responses against less immunogenic self-derived tumour antigens. In contrast, a heterologous prime-boost strategy focuses the secondary immune response on the OV-encoded transgene while generating a less robust primary response against the boosting vector^3,5,6^.

Our group and others have pioneered the use of vesicular stomatitis virus (VSV) as an OV^7–12^. More recently we have screened a subset of rhabdoviruses to identify a candidate with oncolytic properties superior to VSV and free of pathogenic properties in agricultural animals. This led to the identification of Maraba virus (MG1); a rhabdovirus that is exquisitely tropic for both human and murine cancer cells with inherent type I interferon signaling defects^13^. MG1 has only been found in insects, and a serological survey of mammals endemic to the region in Brazil, where the insect originated, failed to identify an animal reservoir and indicated fewer than 1% of humans with a serological response^14^. Analysis of *Rhabdoviridae* from South America identified MG1 to be genetically and serologically distinct from other members of the group and it is non-pathogenic in mammals^15^.

Our preclinical research in mice demonstrated that OV therapy and tumour vaccination could be synergized when using an OV as an intravenously-administered booster vaccine^3,16^. We have shown that an attenuated recombinant MG1 is an excellent vector to boost primary tumour-specific immune responses^17^. In tumour-bearing mice, intravenous injection of oncolytic rhabdoviruses directly engaged antigen-presenting cells to induce massive anti-tumour immune responses^2–4,17^. This immunological benefit occurred concomitantly with infection of tumour cells, leading to acute viral oncolysis and tumour de-bulking. In this setting, the numbers of tumour-infiltrating cancer-specific T cells were increased^3^ and mice could be cured of aggressive melanomas in lungs and brains^3,18^. Our goal is to advance this therapy into cancer patients.

Translation of cancer treatments from inbred mice into patients is challenging because these highly controlled models usually do not accurately recapitulate genetic diversity, etiology or pathology of natural disease. In contrast, companion animals such as cats have similar genetic diversity across breeds and spontaneously develop cancers that are remarkably similar to those in humans^19^. Indeed, recognition of the utility of studying spontaneous cancers in companion animals was demonstrated when the National Cancer Institute in the USA established a program in Comparative Oncology in 2003^20^. This led to a clinical trials network called the Comparative Oncology Trials Consortium, of which the Ontario Veterinary College (OVC) at the University of Guelph, Ontario, Canada, is a member site. The only previous report of testing an oncolytic rhabdovirus in companion animals evaluated the safety of an interferon-β-expressing VSV as a monotherapy in dogs^21,22^. Here, we report the results from studies designed to support translation of oncolytic vaccines into clinical trials. Specifically, we evaluated the safety of a heterologous prime-boost vaccination strategy with a replication-competent, attenuated MG1-vectored booster vaccine in cats. The aims were to characterize a maximum tolerated dose of MG1, identify potential adverse events, and assess routes and duration of viral shedding in healthy, purpose-bred, immunocompetent cats.

## Results

### Pilot study

**Figure 1:**
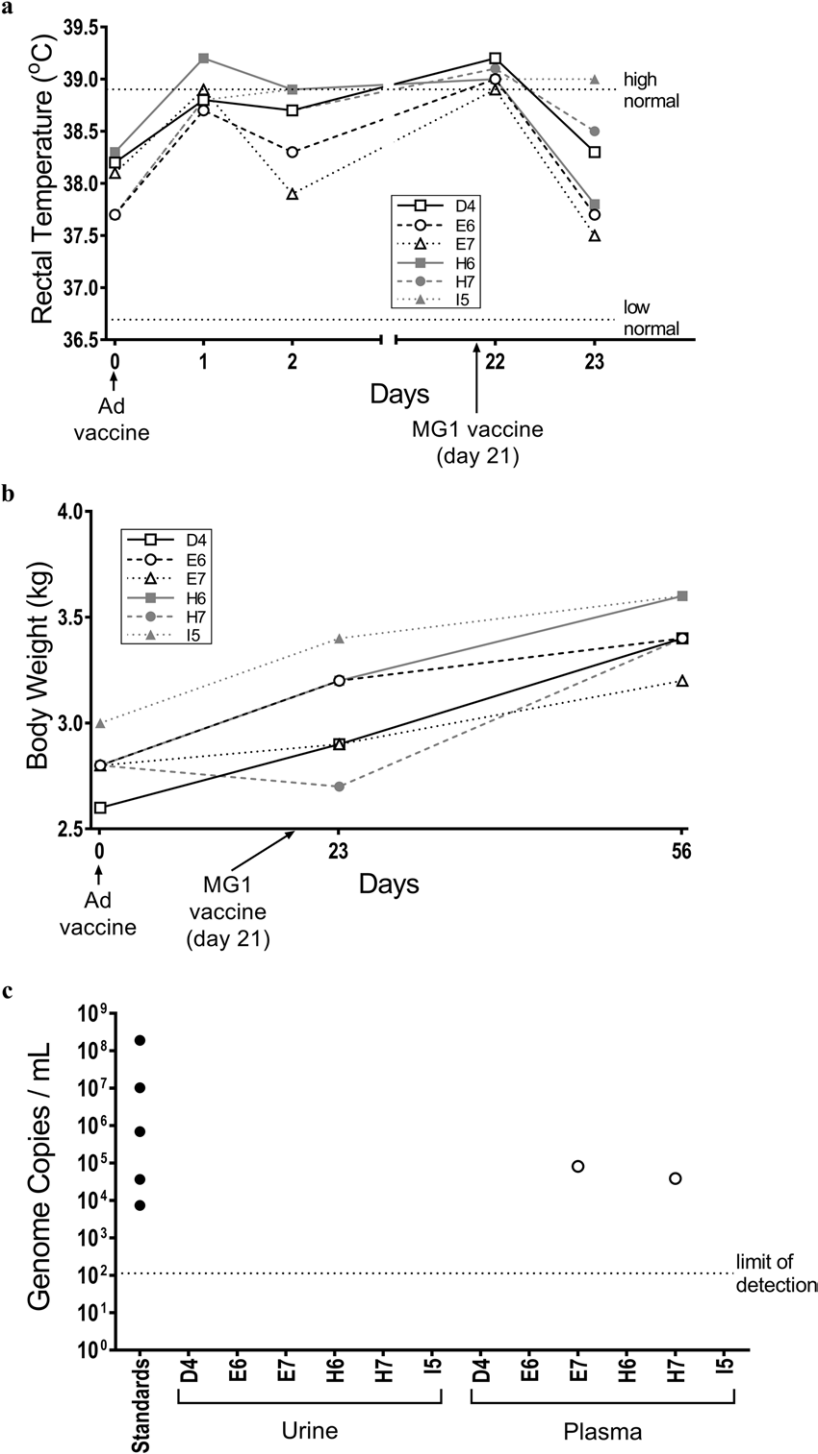
Results of a pilot study to assess the safety of Maraba virus in cats. Six healthy purpose-bred research cats were vaccinated intramuscularly with 1 × 10^10^ pfu of an E1/E3-deleted replication-deficient recombinant human serotype 5 adenovirus (Ad) expressing the melanoma-associated antigen human dopachrome tautomerase (hDCT) on day 0. On day 21 cats were boosted by intravenous infusion of 2 × 10^9^ (cats #H6 and H7), 2 × 10^10^ (cats #I5 and D4) or 2 × 10^11^ (cats #E6 and E7) pfu of a replication-competent Maraba virus (MG1) expressing hDCT. (**a**) Rectal temperatures and (**b**) weights were monitored. (**c**) Urine and plasma samples acquired on day 23 of the study (*i*.*e*. two days post-boost) were assessed for the presence of Maraba virus genomes by quantitative RT-PCR. Data values for each individual cat are shown.

For an initial assessment of the safety of MG1-vectored cancer vaccines, a pilot heterologous prime-boost vaccine study was conducted in six healthy outbred cats. The primary vaccine consisted of a single intramuscular (IM) dose (1 × 10^10^ plaque-forming units [pfu]) of an E1/E3-deleted, replication-deficient recombinant human serotype 5 adenovirus (Ad5) with a transgene encoding human dopachrome tautomerase (huDCT; a melanoma-associated antigen). This was followed 21 days later by one of three different intravenous (IV) doses (2 × 10^9^, 2 × 10^10^ or 2 × 10^11^ pfu; two cats were tested per dose) of a MG1-huDCT booster vaccine. Respiration, rectal temperature, hair coat, food intake, fecal output, body weight, and injection site changes were monitored. No obvious adverse signs were observed. Compared to baseline temperatures preceding the administration of Ad5-huDCT, which were in the normal range for cats (36.7–38.9 °C^23^), body temperatures increased from an average of 38.0 °C to 38.9 °C (Fig. 1a; p = 0.0005; one-way analysis of variance [ANOVA]), with pyrexia induced in one cat at 24 hours post-Ad5. The MG1-huDCT booster vaccine promoted a mild increase in body temperatures, from a mean of 38.0 °C to 39.0 °C at 24 hours post-infusion compared to before treatment (Fig. 1a; p = 0.0015), with five of six cats becoming pyrexic^23^. Average body temperatures returned to pre-treatment levels by 48 hours post-vaccination. Five of six cats had no evidence of a decrease in body weight over the course of this study, although minimal timepoints were evaluated (Fig. 1b). Cat #H7 was the only exception (Fig. 1b). It received a dose of 2 × 10^9^ pfu of MG1 and had a slightly lower body weight (*i*.*e*. a decrease of 0.1 kg) one day post-MG1-huDCT as compared to prior to receiving the primary Ad5 vaccine. However, its body weight increased to the mean for the group by the end of the study. Eighty-one days after treatment with MG1, cats were sedated and euthanized to perform necropsies and histopathologic evaluation of tissues. Neither gross nor histologic lesions suggestive of disease or adverse effects were identified. Splenic lymphoid hyperplasia, an expected outcome of vaccination, was observed in all six cats. Plasma and urine samples were collected two days after vaccination with MG1-huDCT to assess potential shedding of the virus. No MG1 genomes were found in urine samples, while two of six plasma samples were positive (Fig. 1c). These two samples (from cats #E7 and H7) were further analyzed with a viral titration assay, which did not yield any plaques. Having gained confidence that MG1 was well-tolerated by cats, with no obvious adverse events, a more intensive study was designed to facilitate a greater number of assessments at more timepoints.

### Pre-clinical safety study design

A preclinical safety study was performed under the guidance of the Canadian Food Inspection Agency (CFIA) and the Canadian Centre for Veterinary Biologics (CCVB) to further assess the safety of an Ad5-prime-MG1-boost strategy in five healthy, outbred cats. Guidelines from the CCVB for the assessment of novel vaccines can be found here: http://www.inspection.gc.ca/animals/veterinary-biologics/guidelines-forms/eng/1299160285341/1320704254070. Instead of the huDCT transgene used in the pilot study, the viral vectors used in the pre-clinical study were engineered to encode the human placenta specific 1 (huPLAC1) transgene, which is also a candidate tumour-associated antigen. This study was designed to rigorously assess potential shedding of MG1 post-vaccination. The vaccination schedule was similar to the pilot study (*i*.*e*. a 21-day interval between priming with 1 × 10^10^ pfu of Ad5 and boosting with MG1) except that all cats received a higher dose of the MG1 booster vaccine (2.5 × 10^11^ pfu) since 2 × 10^11^ pfu had been very well-tolerated in the pilot study. Cats were euthanized 18 days after receiving the booster vaccination.

### Vaccinating with MG1 caused salivation during administration followed by acute, transient, mild pyrexia

**Figure 2:**
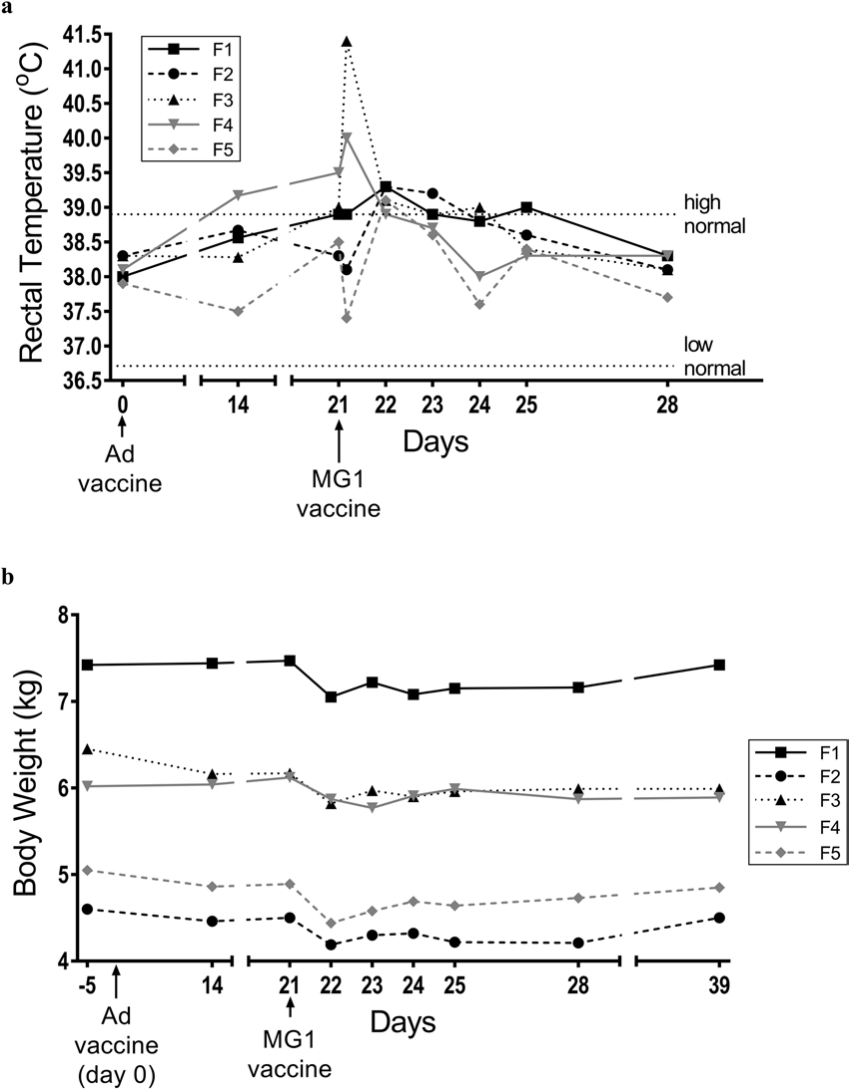
The effect of Maraba virus on body temperatures and weights. Five healthy purpose-bred research cats were vaccinated intramuscularly with 1 × 10^10^ pfu of an E1/E3-deleted replication-deficient recombinant human serotype 5 adenovirus (Ad) expressing the tumour-associated antigen human placenta specific 1 (hPLAC1) on day 0. On day 21 cats were boosted by intravenous infusion of 2.5 × 10^11^ pfu of a replication-competent Maraba virus (MG1) expressing hPLAC1. (**a**) Rectal temperatures and (**b**) weights were monitored. Data values for each individual cat are shown.

A common response to viral infections is the induction of pyrexia^24^. Therefore, we monitored the rectal temperatures of cats treated with MG1. Changes in body temperatures at four hours post-vaccination with MG1-huPLAC1 were variable, with one of five cats developing moderate pyrexia, one developing mild pyrexia, two remaining relatively unchanged and one transiently decreasing below normal (Fig. 2a). The temperatures of all cats returned to normal by 21 hours after boosting with MG1-huPLAC1. Notably, we observed an increase in salivation and lip-smacking, likely attributable to nausea, during the intravenous administration of MG1 in three cats. This subsided within one hour post-treatment.

### MG1 caused mild acute weight loss

A previous study that characterized the safety of oncolytic VSV in dogs reported the induction of transient flu-like signs^22^, which can be associated with weight loss; so we included this as a parameter when monitoring cats. For approximately 24 hours after receiving the MG1-huPLAC1 booster vaccine, all five cats had inappetence based on observations of reduced food and water intake. As a result, all cats had an average weight loss of 0.37 kg at 24 hours post-MG1-huPLAC1 (p = 0.0001) that remained evident up to four days post-vaccination. Body weights had returned to normal by eighteen days post-MG1 (Fig. 2b).

### MG1 caused transient leukopenia, lymphopenia, thrombocytopenia, and neutrophilia in some cats

**Figure 3:**
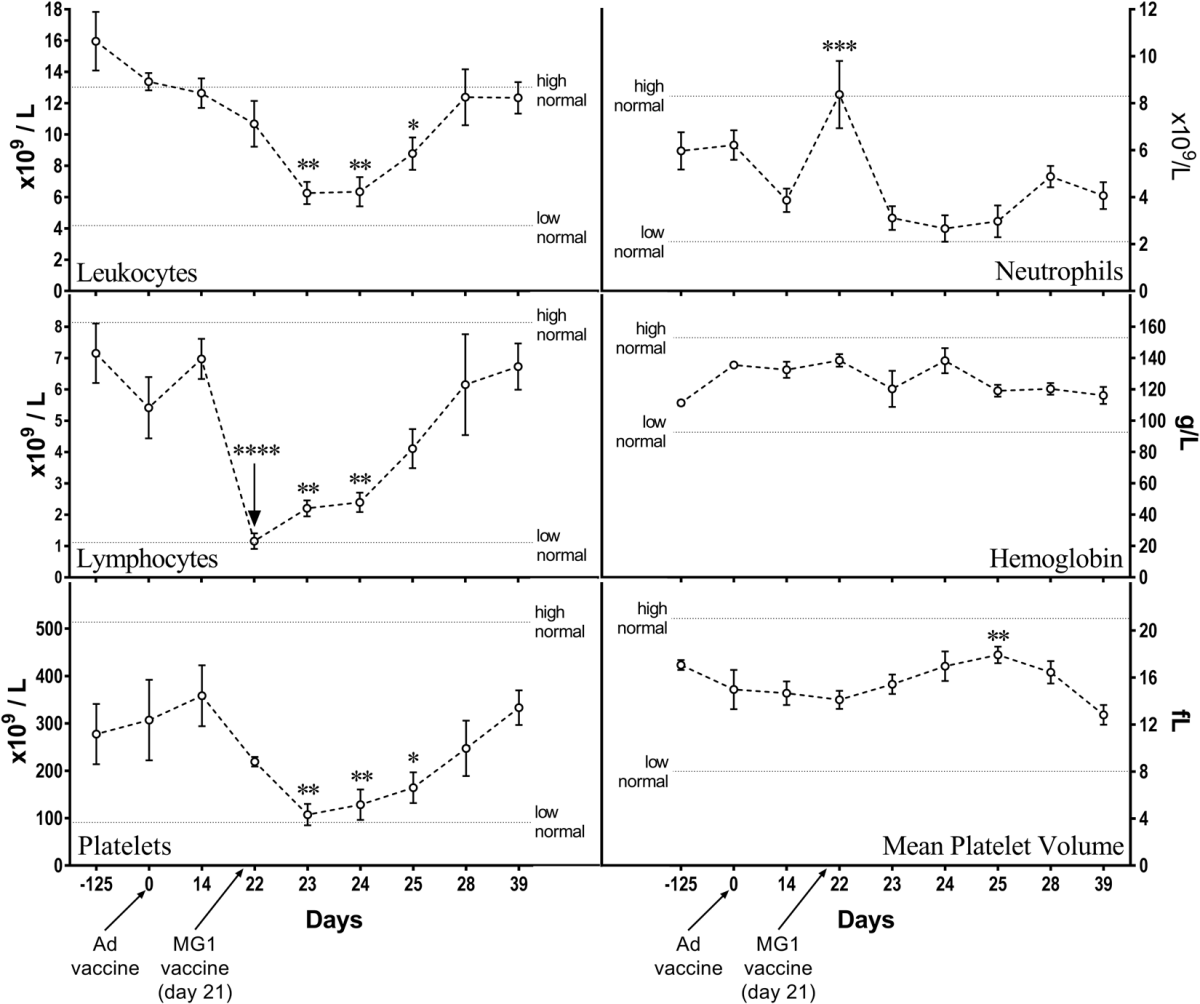
Complete blood count data from cats treated with Maraba virus. Five healthy purpose-bred research cats were vaccinated intramuscularly with 1 × 10^10^ pfu of an E1/E3-deleted replication-deficient recombinant human serotype 5 adenovirus (Ad) expressing the tumour-associated antigen human placenta specific 1 (hPLAC1) on day 0. On day 21 cats were boosted by intravenous infusion of 2.5 × 10^11^ pfu of a replication-competent Maraba virus (MG1) expressing hPLAC1. Complete blood counts were monitored. Data shown represent the numbers of blood-derived leukocytes (upper left panel), neutrophils (upper right panel) and lymphocytes (middle left panel), the concentration of hemoglobin (middle right panel), the number of platelets (lower left panel) and mean platelet volume (lower right panel). Means and standard errors are shown. No significant differences due to treatment with adenovirus were found, so data following administration of Maraba virus were analyzed by one-way analysis of variance and compared to pre-booster levels on day 14 (*p < 0.05, **p < 0.01, ***p < 0.001, ****p < 0.0001).

Since we previously observed an induction of lymphopenia in pre-clinical testing of an oncolytic vaccine^18^, complete blood counts were performed on cats before and after treatment with both Ad5 and MG1. Complete blood counts were unaffected by the administration of Ad5, based on comparisons between blood samples taken immediately prior to vaccination and those acquired 14 days later. In contrast, when using the samples taken 14 days post-Ad5 (*i*.*e*. seven days prior to treatment with MG1) as a baseline, MG1 induced numerous, transient changes in blood. Specifically, the numbers of leukocytes decreased significantly two, three, and four days post-MG1-huPLAC1, which was largely due to fewer circulating lymphocytes (Fig. 3, upper left and middle left panels). Notably, the reduction in leukocytes was not observed one day post-MG1 because the lower numbers of lymphocytes were offset by a significant increase in the number of neutrophils (Fig. 3, upper right panel), which only lasted approximately 24 hours. Leukocyte numbers returned to normal by seven days post-MG1. Between days one to four post-MG1, four cats (#F2, F3, F4, F5) experienced lymphopenia, two cats (#F3, F5) had leukopenia and one cat (#F5) had neutrophilia. A significant reduction in the numbers of platelets were observed on days two, three, and four post-MG1, with a concomitant increase in mean platelet volume four days after treatment with MG1 (Fig. 3, lower left and lower right panels). Out of the five cats, two (#F1, F5) experienced thrombocytopenia. Platelet characteristics returned to pre-treatment values by seven days post-MG1.

### Cats boosted with MG1 were neither viremic nor shed the virus

**Figure 4:**
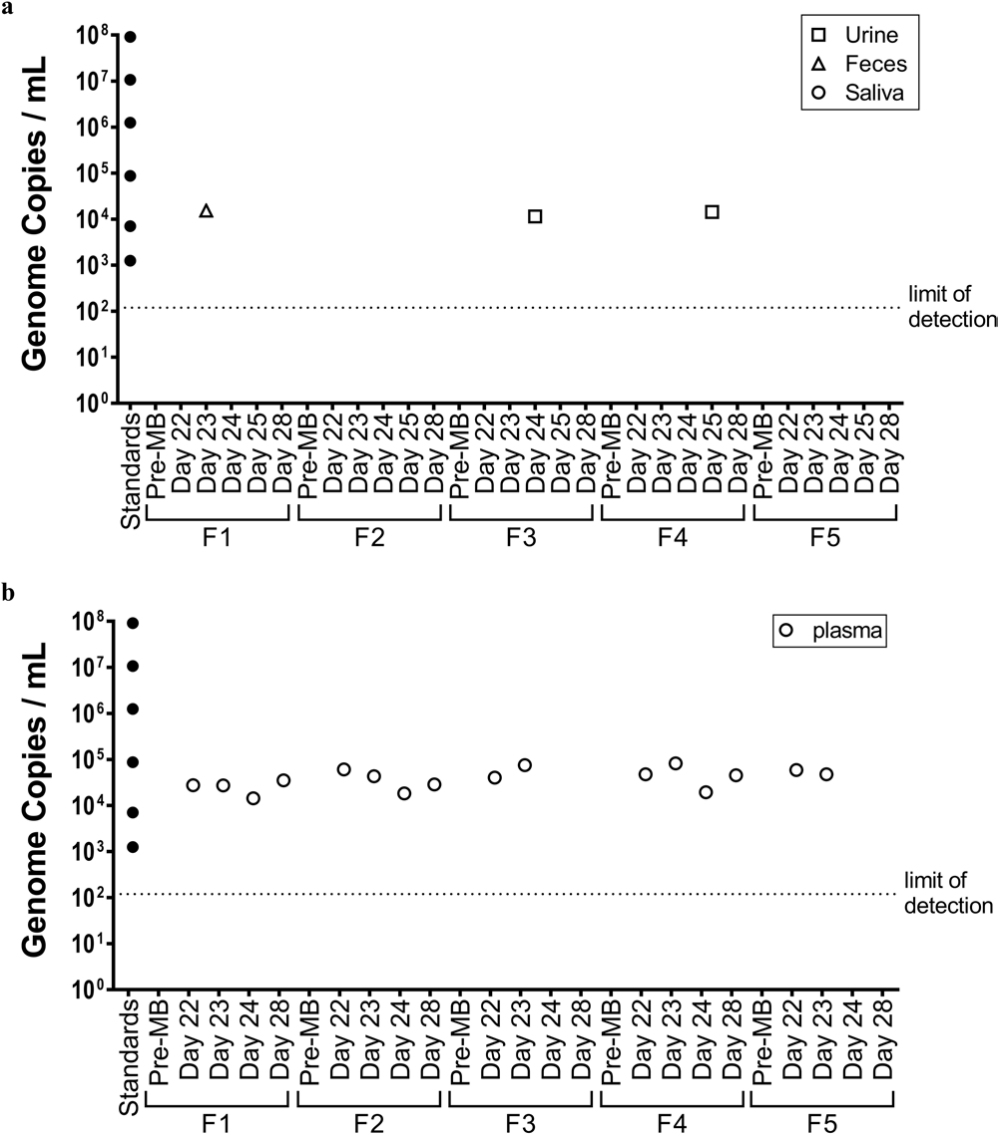
Assessment of tissues for the presence of Maraba viral genomes. Five healthy purpose-bred research cats were vaccinated intramuscularly with 1 × 10^10^ pfu of an E1/E3-deleted replication-deficient recombinant human serotype 5 adenovirus expressing the tumour-associated antigen human placenta specific 1 (hPLAC1) on day 0. On day 21 cats were boosted by intravenous infusion of 2.5 × 10^11^ pfu of a replication-competent Maraba virus expressing hPLAC1. (**a**) Urine, feces, saliva and (**b**) plasma samples were taken one, two, three, four and seven days post-boost for quantification of Maraba viral genomes by quantitative reverse transcriptase-polymerase chain reaction. Data values for each individual cat are shown.

Assessment of duration of shedding of a viral vector is an important consideration for patient management and infection control guidelines. Therefore, potential shedding of MG1 was evaluated by quantifying viral genomes by reverse transcriptase-polymerase chain reaction (RT-PCR) in urine, feces, and saliva (Fig. 4a). After administration of MG1-huPLAC1, ~10^4^ viral genomes were detected in one urine sample each from a cat (#F3) three days post-MG1 and a cat (#F4) four days post-MG1, and in one fecal sample from a cat (#F1) two days post-MG1. MG1-derived genomes were not detected in any saliva samples. Replication-competent MG1 could not be recovered from any of the urine or fecal samples that contained viral genomes.

Blood samples were also collected from cats to assess viremia post-vaccination with MG1-huPLAC1. On days one and two post-MG1, all cats had between 10^4^ and 10^5^ genome copies in plasma (Fig. 4b). Three days after MG1 vaccination MG1 genomes were detected in plasma from three of five cats. On day 7 post-MG1 vaccination the same three cats had genomes present in plasma. All plasma samples from day seven post-MG1 were tested for the presence of replication-competent viruses using a standard plaque assay. To maximize the sensitivity for detection, plasma was placed directly onto permissive Vero cells, which allows formation of plaques by single infectious particles^25^. No replication-competent viruses were recovered from the plasma samples.

### Vaccination with MG1 was non-pathogenic

**Figure 5:**
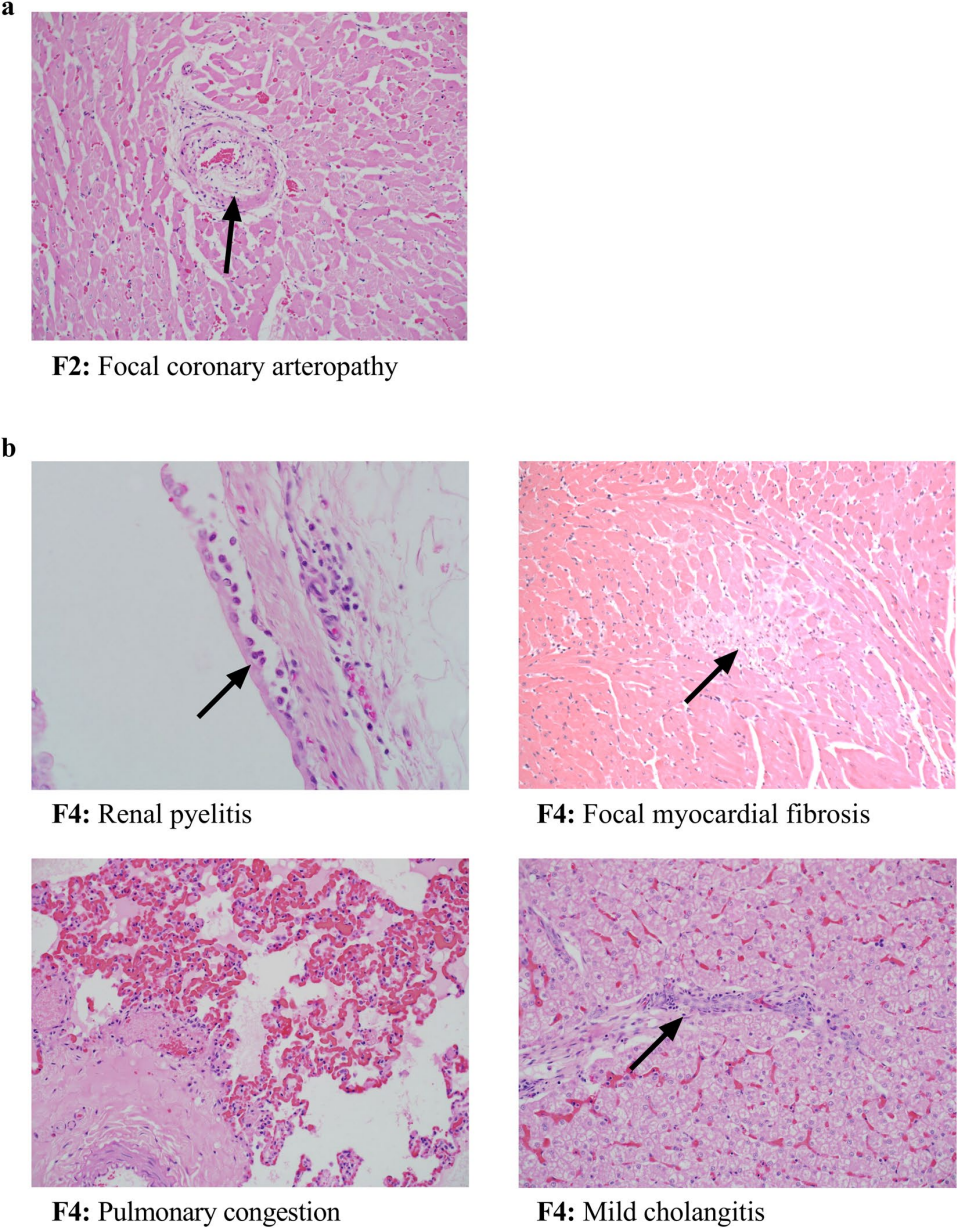
Post-mortem lesions of unknown etiology from cats treated with Maraba virus. Five healthy purpose-bred research cats were vaccinated intramuscularly with 1 × 10^10^ pfu of an E1/E3-deleted replication-deficient recombinant human serotype 5 adenovirus expressing the tumour-associated antigen human placenta specific 1 (hPLAC1) on day 0. On day 21 cats were boosted by intravenous infusion of 2.5 × 10^11^ pfu of a replication-competent Maraba virus expressing hPLAC1. On day 39 (*i*.*e*. 18 days post-boost), cats were euthanized for post-mortem analysis by a board-certified veterinary pathologist. (**a**) One cat had evidence of focal coronary arteropathy consisting of a partially thickened arterial wall (arrow). (**b**) One other cat had mild renal pyelitis with subepithelial infiltration of neutrophils (arrow, upper left panel), focal myocardial fibrosis (arrow, upper right panel), pulmonary congestion (lower left panel) and mild cholangitis (arrow lower right panel).

At the end of the study (18 days post-treatment with MG1), all five cats appeared healthy, with no clinically relevant signs and were behaving normally. However, to facilitate a comprehensive assessment of possible sub-clinical changes, the cats were sedated and euthanized to perform post-mortem analyses. Histopathological evaluations revealed all five cats had splenic lymphoid hyperplasia and congestion, and three out of five cats also had lymph node hyperplasia. Other findings are summarized in Table 1 and Fig. 5 and included mild myocardial congestion, focal myocardial fibrosis, focal coronary arteropathy, hepatic congestion, mild cholangitis, and mild pyelitis. Note that each lesion was found in only one cat.

**Table 1:** Summary of post-mortem histopathological findings in cats.

Animal ID	Splenic lymphoid hyperplasia	Splenic congestion	Myocardial congestion	Focal myocardial fibrosis	Coronary arteropathy	Renal medullary tubular protein	Hepatic congestion	Mild cholangitis	Mild pyelitis
F1	+	+							
F2	+	+			+	+			
F3	+	+	+				+		
F4	+	+		+				+	+
F5	+	+

### MG1 genomes were detected in spleen, heart, and lung tissues

**Figure 6:**
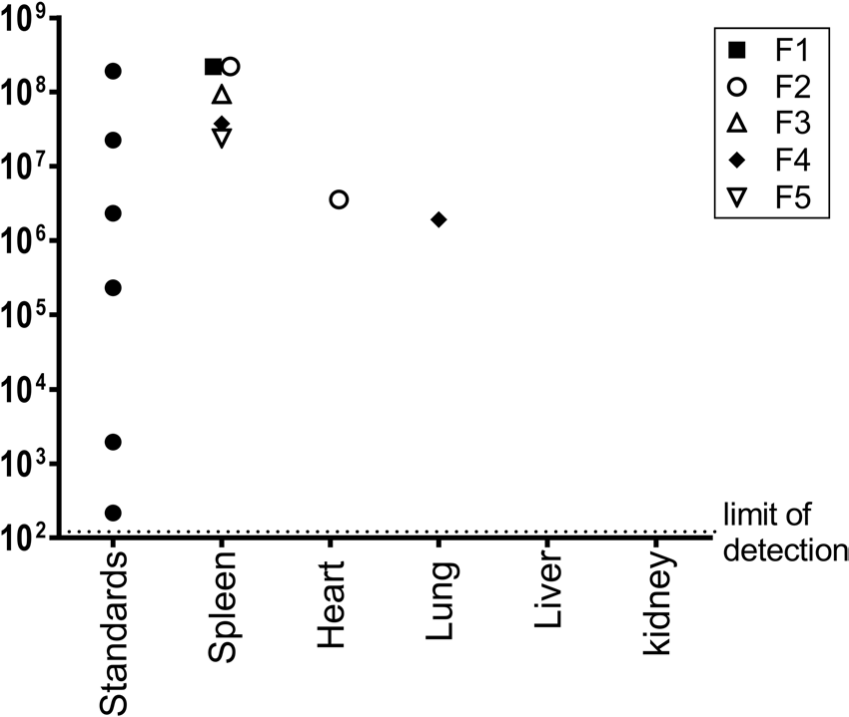
Post-mortem biodistribution of Maraba viral genomes. Five healthy purpose-bred research cats were vaccinated intramuscularly with 1 × 10^10^ pfu of an E1/E3-deleted replication-deficient recombinant human serotype 5 adenovirus expressing the tumour-associated antigen human placenta specific 1 (hPLAC1) on day 0. On day 21 cats were boosted by intravenous infusion of 2.5 × 10^11^ pfu of a replication-competent Maraba virus expressing hPLAC1. On day 39 (*i*.*e*. 18 days post-boost), cats were euthanized for post-mortem quantification of Maraba viral genomes by quantitative RT-PCR. Tissues that were assessed included spleens from all cats, plus tissues with any abnormal lesions, which included the heart of one cat and the lung, liver and kidney of one other cat. Data values for each individual cat are shown.

Tissues with lesions were analyzed by multiplex VSV/MG1 quantitative PCR (qPCR) to investigate for the presence of MG1 genomes. RNA was extracted from the spleens of all cats, heart, liver, lung and kidney tissues of cat #F4, and heart tissue of cat #F2. Between 1 × 10^7^ and 5 × 10^8^ copies of viral genomes were detected in the spleens of all cats (Fig. 6) but subsequent virus titration assays did not yield any plaques. MG1 genomes were detected at lower levels (~10^6^ copies) in the heart and lung of cats #F2 and F4, respectively. Similar to the viral titer assays, spleen, heart and lung tissues also did not yield evidence of the presence of replication-competent MG1.

## Discussion

Cancers in humans and companion animals are strikingly similar, including sites of occurrence, histological and molecular features, metastatic propensity, and responses to treatments; all overlaid on similarly diverse genetic and environmental backgrounds. However, clinical trials designed to test novel viral-vectored biotherapies in comparative oncology are lacking. To date, the majority of studies have tested non-oncolytic adenoviral vectors in dogs and while a few have investigated OVs, these studies have largely been limited to xenograft mouse models and *ex vivo* dog and cat tumour infections demonstrating oncolytic properties^21^. One pre-clinical study reported by Autio *et al*. also demonstrated the safety of an oncolytic vaccinia virus in dogs^26^. Dr. Stephen Russell’s group at the Mayo Clinic (Rochester, Minnesota, USA), was the first to characterize oncolytic VSV as a safe monotherapy in dogs^27^. In our hands, MG1 has proven to be a more oncolytic rhabdovirus than VSV in numerous cancer cell lines^13^. Pre-clinical murine studies by our group and others have shown that MG1 is not only potently oncolytic but when used in a heterologous Ad-prime/MG1-boost strategy, it induces massive tumour-specific T-cell responses^2,3,17^. To begin the process of translating this promising prime-boost vaccine strategy into feline cancer patients, we evaluated the feasibility of using MG1-vectored vaccines in tumour-free purpose-bred research cats.

When administering vaccines IM or IV, there is a common list of adverse reactions clinicians can anticipate for both humans and animals (*e*.*g*. reaction at injection sites, muscle pain/stiffness, pyrexia, reduced body temperature, loss of appetite, headaches, nausea, and vomiting). Flu-like symptoms, especially pyrexia and nausea are reportedly the most common adverse events seen in humans, non-human primates, and dogs receiving oncolytic virotherapies^27–30^. In our cat studies, the effect of MG1 on body temperatures was variable, with some cats experiencing a slight reduction, some a slight increase, and others remaining relatively unchanged (Figs 1a and 2a). Only one cat had its temperature fall below the lower limit of the normal range ( < 37.7 °C), while one cat experienced a mild fever and one experience a moderate fever (*i*.*e*. ≥ 40.0 °C^31^). In all cases, body temperatures normalized within 24 hours post-infusion. Fever, nausea and inappetence can result in weight loss in some patients following vaccination. During the infusion of MG1, we observed signs of nausea (salivation and lip smacking) in three out of the five cats that received the highest dose (2.5 × 10^11^ pfu). This reaction subsided very quickly after infusion. Slight weight loss within 24 hours of receiving MG1 was a common observation (Figs 1b and 2b). The fact that weight loss resolved and weight gain resumed within 48–72 hours post-MG1 suggested the primary cause may have been mild dehydration due to a lack of water intake in the day after treatment. Unlike the previously reported study in which VSV caused oral mucosal lesions in dogs^32^, these lesions were not observed in cats treated with MG1 at timepoints when saliva samples were taken, nor at post-mortem. Overall, no adverse events in the cat studies required clinical intervention. On this basis, we conclude Ad5-prime/MG1-boost vaccination in cats is well-tolerated. It is notable that doses of MG1 up to 2.5 × 10^11^ pfu were well-tolerated by cats with no severe adverse reactions. In contrast a VSV expressing IFN-β in dogs became toxic at 1 × 10^11^ pfu, making it necessary to euthanize a dog due to severe hepatotoxicity and signs of shock^32^. In the latter study, the maximum tolerable dose was deemed to be only 1 × 10^10^ pfu. One reason why the cats in our study may have tolerated such high doses of MG1 is the fact that they received primary vaccines with a different virus (Ad5) targeting the same transgene carried by the MG1; an effect that increased the safety of oncolytic virotherapy in pre-clinical studies, presumably by potentiating the clearance of off-target infections^3^.

Transient changes in blood chemistry have been reported in patients and animals receiving oncolytic viral therapies^27,29,33^. Complete blood count profiling showed that MG1 induced a transient reduction in the number of leukocytes in circulation, which was most apparent with lymphocytes (Fig. 3). Our results are similar to studies in dogs where LeBlanc *et al*. reported transient lymphopenia after treatment with recombinant rhabdovirus VSV-IFNb-NIS (a VSV that expresses interferon-β and the sodium iodide symporter reporter gene). Similar lymphopenic events were described in human patients with primary and metastatic liver cancers treated with oncolytic vaccinia virus JX-594^33^. Viral infections have been shown to induce type I interferons that transiently reduce the circulation of lymphocytes in the blood^34^. Since rhabdoviruses are inducers of type I interferons^9^, this mechanism provides a possible explanation for the lymphopenia observed in cats treated with MG1. In contrast to the effect on lymphocytes, an acute increase in the numbers of neutrophils in blood was observed within 24 hours of cats receiving MG1. Neutrophilia is a common and often protective response to viral infections^35^. Notably, oncolytic rhabdoviruses are known to mediate an acute vascular shutdown in tumours, which is mediated by neutrophils within five to 24 hours post-infusion^36^. Therefore, the observation of acute neutrophilia in cats post-MG1 is in agreement with what has been previously reported. Another important finding was an acute, transient decrease in the number of circulating platelets within the 72 hours of treatment with MG1, resulting in thrombocytopenia in two of five cats receiving the highest dose (2 × 10^11^ pfu). We interpret the increase in mean platelet volume at 96 hours post-MG1 to be a compensatory reaction to the loss of platelets. When a decrease in platelets was found at 24 hours post-infusion, we monitored for abnormal bleeding at jugular needle puncture sites during all subsequent blood draws. In all cases, we did not have any difficulty stopping bleeding, with clot formation occurring within less than 90 seconds of applying mild pressure with gauze to puncture wounds. Acute, mild to moderate thrombocytopenia was previously reported following intravenous infusion of oncolytic Newcastle disease viruses^37^ and vaccinia viruses^33^ into human patients. Thrombocytopenia was reported as a dose-limiting toxicity in mice treated intravenously with VSV^32^. Taken together, the transient changes in blood chemistry we documented in cats treated with the Ad5-prime/MG1-boost vaccination strategy were low-grade adverse events and have been reported by others administering OVs as novel cancer therapies.

When considering the use of recombinant OVs as clinical biotherapies, it is important to determine whether viremia could be induced that could result in shedding of the OV. Given that MG1 is not a mammalian virus, the pharmacokinetics of MG1 in cats has never been reported. Plasma samples from the blood of all 11 cats were analyzed by multiplex qPCR to detect MG1 genomes. On the second day after boosting with MG1, seven cats (64%) had detectable MG1 genomes in their blood (between 10^4^–10^5^ copies; Figs 1c and 4b). Further testing of two of these seven samples from 48 hours post-MG1 using a standard plaque assay failed to yield any replication-competent viruses. Of these seven cats, three of them remained positive for MG1 genomes in their blood up to seven days post-MG1 (10^4^–10^5^ copies/mL). These plasma samples were screened by the plaque assay and like the blood samples from cats analyzed at day two post-MG1, none of them contained replication-competent viruses. Our data are consistent with LeBlanc *et al*. who detected as high as ~1 × 10^6^ genome copies/μg of RNA 24 hours post-VSV-IFNb-NIS vaccination and the persistence of viral genomes in plasma up to 10 days post-treatment^27^.

Biological samples were also collected during the course of these cat studies to assess the shedding potential of MG1. Urine was collected from all 11 cats. Fecal and saliva swab samples were also collected for the five cats in the preclinical study. Multiplex qPCR detected MG1 genomes in two urine samples (~10^5^ and ~10^4^ copies at days three and four post–MG1, respectively). All saliva samples were found to be negative and one fecal sample was positive (~10^4^ genome copies on day two post-MG1). Like the plasma samples, sensitive viral titre assays failed to detect replication-competent viruses in any genome-positive urine or fecal samples. Our cat studies show for the first time, that a MG1-booster vaccine does not potentiate chronic viremia or lead to the shedding of replication-competent viruses from cats treated with high doses up to 2.5 × 10^11^ pfu.

Studies in both dogs and non-human primates have reported autopsy results showing that oncolytic viral therapies are non-pathogenic to normal tissues^26,27,38,39^. Autopsy of the cats in this study revealed that a few tissues had lesions of undetermined etiology. The spleens of all cats showed lymphoid hyperplasia and some lymph node involvement that was a possible reactive change associated with immune stimulation, such as vaccination with MG1. Multiplex qPCR confirmed the presence of MG1 genomes in spleens from all cats (up to ~5 × 10^8^ copies) but no replication-competent viruses were found. Association of MG1 with the spleen was not an unexpected finding since OV genomes have been consistently isolated from the spleens of treated mice, dogs and non-human primates^22,26,40–42^. One cat with a focal coronary arteropathy in a single blood vessel of the heart was positive for MG1 genomes (~10^6^ copies) and another cat with mild cholangitis and lung congestion was positive (~10^6^ copies). Importantly, no replication-competent viruses were detected in any of these tissues. As such, it is impossible to conclude whether the lesions that were found were incidental or due to treatment with the Ad5-prime/MG1-booster vaccines. Nonetheless, current standard of care cancer treatments have substantial harmful side effects and none of the cats at the end of these studies showed any clinically relevant signs. Therefore, we did not consider any post-mortem lesions to represent dose-limiting toxicities. Indeed, others have reported unexpected adverse events in dogs treated with VSV for which the etiology was difficult to establish. These included prolongation of partial thromboplastin time, development of bacterial urinary tract infection, and scrotal dermatitis^32^. We speculate that the presence of MG1-derived genomes but an absence of replication-competent viruses in tissues may be due to retention of virus-derived RNA following processing of viral particles in antigen-presenting cells.

This study represents the first description of clinical signs, blood cell profiles, viremia, viral shedding, and histopathology of a systemically administered oncolytic MG1 in cats. Although MG1 is not considered a mammalian pathogen and the recombinant version of the virus used is reported to preferentially replicate in tumours^13^, we have taken the first step towards translating its use into clinical trials by clearly demonstrating that a heterologous Ad5-prime/MG1-boost vaccination strategy was well-tolerated in cats; even at higher doses (up to 2.5 × 10^11^ pfu) than what were used previously for other rhabdoviruses, like VSV, in larger dogs^27^. Like reports on other OV-based therapies, MG1 booster vaccines can cause transient flu-like symptoms, but they are non-toxic, non-pathogenic, and do not result in shedding of replication-competent viruses. These results presented here support the use of Ad5-prime/MG1-booster vaccination as a promising, novel therapy for testing in the context of veterinary clinical trials.

## Methods

### Outbred Cats

All cats used in these studies were purpose-bred and specific pathogen-free (Liberty Research, Waverly, NY, USA). They received food and water *ad libitum*, were group-housed in an environmentally controlled room with enrichment that included daily play-time with animal care technicians. Experiments were conducted in the containment level-2 Isolation Unit in the Ontario Veterinary College (University of Guelph, Guelph, Ontario, Canada). Studies complied with Canadian Council on Animal Care guidelines and were approved by the University of Guelph’s Animal Care Committee under Animal Utilization Protocol #1912. An initial pilot study was conducted with six female cats, which were assigned the following identification numbers: D4, E6, E7, H6, H7 and I5. This was followed by a more extensive pre-clinical study that utilized five male cats assigned the numbers F1 through to F5. The numbers of cats used were based on the minimum required by the Canadian federal government agencies that provided oversight for these studies (*i*.*e*. CFIA and CCVB). All cats were monitored daily for the duration of each study for any evidence of clinically-relevant signs.

### Viral Vaccine Vectors

The huDCT transgene encoded the full-length human melanoma antigen DCT. The replication-deficient adenovirus vector was based on an E1/E3-deleted human serotype 5 and production of recombinant Ad5-huDCT has been described previously^43^. Recombinant MG1 was generated by hDCT transgene insertion between the genes encoding the glycoprotein (G) and large structural protein (L) of the attenuated MG1 strain^13^ and has been described previously^17^.

The huPLAC1 transgene encoded the full-length huPLAC1 protein and was amplified from A549 cell-derived RNA (ATCC, CRM-CCL-185) using the following RT-PCR primers: GCGAATTCGCCACCATGAAAGTTTTTAAGTTCATA (forward) and GAAGCTTTCAACATGGACCCAATCATAT (reverse). These primers contained EcoRI and HindIII restriction enzyme cloning sites, respectively. The RT-PCR product was blunt-end cloned into the plasmid pJET (ThermoFisher Scientific, Burlington, ON, Canada, product #K123) and then shuttled into a pcDNA3 expression vector (Thermo Fisher Scientific, product #V790-20). The recombinant Ad and MG1 vaccine vectors were generated as previously reported^44^.

### Vaccine Production

The Robert E. Fitzhenry Vector Laboratory at McMaster University (Hamilton, Ontario, Canada) is a Good Manufacturing Practice-level adenovirus vector production facility that adheres to Health Canada guidelines for phase I/II clinical trials (https://www.canada.ca/en/health-canada/services/drugs-health-products/compliance-enforcement/good-manufacturing-practices/guidance-documents/good-manufacturing-practices-guidelines-2009-edition-version-2-0001.html). The facility also has a process development room that was used for pilot-scale batches of the Ad5 vaccine used in the studies described here. The master cell bank used to produce the Ad5-huDCT and huPLAC1 vaccines was 293SF-3F6 (Vector Lab #MCB-04).

Maraba vaccine vectors were produced in a dedicated space under Good Laboratory Practice (https://www.canada.ca/en/health-canada/services/consumer-product-safety/reports-publications/pesticides-pest-management/policies-guidelines/regulatory-directive/1998/good-laboratory-practice-dir98-01.html) and Good Manufacturing Practice guidelines. The cells used to produce MG1-hDCT and MG1-hPLAC1 were 293 T (ATCC, Manassas, VA, USA, product #ASC-4900). Briefly, confluent cell layers were infected with MG1-huDCT or MG1-huPLAC1 at a multiplicity of infection of 0.01. Infected cells and supernatants were harvested, clarified using a 0.22 μm filter, pelleted by ultracentrifugation at 10,000 rpm in a TY-19 rotor (Beckman Coulter Life Sciences, Indianapolis, IN, USA), resuspended in 1 ml of phosphate-buffered saline (PBS) and purified by banding in a 4–20% sucrose gradient in an ultracentrifuge at 24,000 rpm using a SW41 Ti rotor (Beckman Coulter Life Sciences). Purified viruses were dialyzed overnight in PBS using a dialysis cassette with a 20 kDa pore size.

### Administration of Vaccines to Cats

Doses (1 × 10^10^ pfu in 200 μL) of primary Ad5-vectored vaccines were split into two equal volumes (100 μL) that were delivered intramuscularly into each hamstring. The rationale for splitting the primary vaccine dose was to engage two different lymphatic drainage regions.

Maraba virus-vectored booster vaccines were delivered intravenously through a catheter inserted into the cephalic vein. In the pilot study, the doses that were tested included 2 × 10^9^, 2 × 10^10^ and 2 × 10^11^ pfu per cat (two cats tested per dose). Note that the starting dose (2 × 10^9^) was based on the maximum tolerated dose identified for mice in our previous studies. Because 2 × 10^11^ pfu of MG1 was well-tolerated, a dose of 2.5 × 10^11^ pfu was used for all five cats in the follow-up pre-clinical experiment. Cats were sedated using butorphanol (200 μg/kg) and dexmedetomidine (20 μg/kg). The MG1 was diluted in 20 ml of PBS and administered with an infusion pump over 20 min. Cats were recovered from sedation with atipamazole and monitored closely daily for seven consecutive days post-injection for any evidence of reactions to the infusion of MG1.

### Assessment of Clinical Signs

Body temperatures (rectal) and weights were measured at defined timepoints, and blood samples (jugular vein) were collected during both cat studies to characterize the effects of the MG1 vaccine on the maintenance of hydration and appetite, and the potential for induction of pyrexia and/or changes in the numbers of blood-derived cells (complete blood counts were performed by the Animal Health Laboratory, University of Guelph). Upper and lower normal limits for each blood-derived parameter were shown on each graph and these reference intervals represent the central 95% of values from a reference population generated by the Animal Health Laboratory. Injection sites, quality of hair coats, physiologic behaviors such as grooming, food intake, fecal output and respiratory rates were monitored throughout the study.

### Histopathological Assessments

Fifteen tissues (lung, brain stem, cerebellum, frontal lobe, occipital lobe, temporal lobe, heart, inguinal node, spleen, liver, kidney, bladder, adrenal gland, small intestine, large intestine) were collected from each cat after euthanasia, fixed in 10% formalin for 24–48 hours, washed in 70% ethanol and transferred into PBS prior to being embedded in paraffin, sectioned and then stained with hematoxylin and eosin and reviewed microscopically by a board-certified veterinary anatomic pathologist. Tissues with lesions of unknown etiology were tested for the presence of MG1 genomes by multiplex qPCR.

### Plaque Assay for Quantifying Replication-Competent Maraba Virus

For each sample tested, ten 60 mm plates of highly permissive Vero cells (ATCC; CCL81) were grown to a confluent monolayer at 37 °C/5% CO_2_ in Eagle’s Minimum Essential Medium containing 10% fetal bovine serum (Fisher Scientific, Canada). Five 1:10 serial dilutions of each sample were tested in duplicate. To maximize sensitivity of the assay, the initial concentration tested was 100% (neat). After removal of medium from each plate, 100 μl of each sample was added. Cells were rocked to ensure even distribution of the virus and then transferred to an incubator for 45 min., with the plates rocked thoroughly every 10 minutes. After 45 mins., each plate of cells received 3 mL of an agarose overlay (1:1 mixture of low melting point agarose + 2X fetal bovine serum-containing medium; Fisher Scientific) that was liquefied at 42 °C. Note: 1 tube is enough for 15–16 titer plates. Mix 25 ml of 2X medium with FBS + 25 ml of molten 1% agarose to get a final volume of 50 ml. After allowing the agarose to harden for 10 minutes at room temperature, places were returned to the incubator for 30 hrs. For samples that did not appear to have plaques, the assay was repeated, with the total incubation time extended to 48 hrs and the incorporation of staining with crystal violet (Fisher Scientific) to ensure an absence of plaques. Virus titers were calculated as plaque-forming units (pfu)/mL.

### qPCR for Viral Shedding

Samples were collected from cats at specific times pre- and post-vaccination to determine whether MG1 was shed in urine (cystocentesis samples), feces (anal swabs) or saliva (oral swabs), or was retained in plasma. Samples (200 μL) were spiked with 1 × 10^5^ pfu of a VSV-vectored reference virus (*i*.*e*. a VSV that expresses enhanced green fluorescent protein that has been described elsewhere^8^) and RNA was extracted following instructions of the PureLink Viral RNA/DNA mini kit (Thermo Fisher Scientific, product #12280050). Nucleic acids were re-suspended in 35 μL of water.

Sample RNA (1 μL) was added to a qPCR protocol that used a TaqMan One-Step RT-PCR kit (Life Technologies, Carlsbad, CA, USA, product #4309169) in combination with primers and fluorescent probe sets to amplify and quantify MG1 and VSV viral genomes in the samples. The MG1 primer and probe sequences were GGTGATGGGCAGACTATGAAA (forward), CCTAAGGCCAAGAAACAAAAGAG (reverse), 56-FAM/CCTCGATCAAGAGTGTTTGAACCCTGT/3IABkFQ (probe). The VSV primer and probe sequences used were GATAGTACCGGAGGATTGACGACTA (forward), TCAAACCATCCGAGCCATTC (reverse), 5TET/TGCACCGCC/Zen/ACAAGGCAGAGA/3IABkFQ (probe). The qPCR amplification program consisted of 30 min at 48 °C, 10 min 95 °C and 50 cycles of 15 sec at 95 °C, 1 min at 60 °C. Reactions were performed in a 7500 Real-Time PCR machine (Thermo Fisher Scientific).

Viral genome copies in samples were quantified relative to the internal VSV reference signal and then compared to a MG1 standard curve amplified from a series of dilutions ranging from 1 × 10^8^ to 1 × 10^3^ pfu of a reference MG1 that expresses enhanced green fluorescent protein^13^. Each MG1 sample was spiked with the internal control VSV for standardization (1 × 10^5^ pfu). This multiplex MG1/VSV qPCR assay was used to quantify MG1 genomes in samples taken from cats post-vaccination. The limit of detection for this assay was 29 genome copies.

### Data analysis

Graphing and statistical analyses of data were performed with GraphPad Prism version 7.01 (GraphPad Software, Inc., La Jolla, California, USA). Most graphs show data points from individual cats. Data derived from complete blood counts were shown as group means with standard error bars. Differences between means were assessed using a one-way analysis of variance, with body weights first being normalized to pre-vaccination levels. Differences were deemed to be statistically significant if p-values were less than or equal to 0.05.

### Data availability

The datasets generated during and/or analysed during the current study are available from the corresponding author on reasonable request.
